# Modification of Silver Nanowire Coatings with Intense Pulsed Ion Beam for Transparent Heaters

**DOI:** 10.3390/nano10112153

**Published:** 2020-10-29

**Authors:** Marat Kaikanov, Bauyrzhan Amanzhulov, Gulzat Demeuova, Gulnur Akhtanova, Farabi Bozheyev, Aidar Kemelbay, Alexander Tikhonov

**Affiliations:** 1 Physics Department, School of Sciences and Humanities, Nazarbayev University, Nur-Sultan 010000, Kazakhstan; gulnur.akhtanova@nu.edu.kz (G.A.); farabi.bozheyev@nu.edu.kz (F.B.); aidar.kemelbay@nu.edu.kz (A.K.); atikhonov@nu.edu.kz (A.T.); 2 National Laboratory Astana, Nazarbayev University, Nur-Sultan 010000, Kazakhstan; bauyrzhan.amanzhulov@nu.edu.kz (B.A.); gdemeuova@nu.edu.kz (G.D.)

**Keywords:** silver nanowires, transparent heater, intense pulsed ion beam, protective layer

## Abstract

In this report, an improvement of the electrical performance and stability of a silver nanowire (AgNW) transparent conductive coating (TCC) is presented. The TCC stability against oxidation is achieved by coating the AgNWs with a polyvinyl alcohol (PVA) layer. As a result, a UV/ozone treatment has not affected the morphology of the AgNWs network and the PVA protection layer, unlike non-passivated TCC, which showed severe degradation. The irradiation with an intense pulsed ion beam (IPIB) of 200 ns duration and a current density of 30 A/cm^2^ is used to increase the conductivity of the AgNWs network without degradation of the temperature-resistant PVA coating and decrease in the TCC transparency. Simulations have shown that, although the sample temperature reaches high values, the ultra-high heating and cooling rates, together with local annealing, enable the delicate thermal processing. The developed coatings and irradiation strategies are used to prepare and enhance the performance of AgNW-based transparent heaters. A single irradiation pulse increases the operating temperature of the transparent heater from 92 to 160 °C at a technologically relevant voltage of 12 V. The proposed technique shows a great promise in super-fast, low-temperature annealing of devices with temperature-sensitive components.

## 1. Introduction

Transparent conductive coatings (TCCs) are important building blocks in modern optoelectronic devices, such as touch panels, solar cells, thin film heaters, and others [1,2,3]. Indium-doped tin oxide (ITO) is a widely used TCC material and an industry standard due to its good optoelectronic properties. However, ITO application is limited by the high fabrication cost, while its brittleness makes it unsuitable for flexible electronics. Various alternative materials, such as carbon nanotubes [4,5], graphene [6,7], conductive polymers [8,9], and metallic nanowires [10,11] are being explored, among which silver nanowires (AgNWs) have attracted special attention because of their high electrical conductivity, high transparency, and excellent mechanical properties [1,2,3,12,13,14]. The most commonly used method of AgNWs synthesis is the polyol method, which is a simple, yet highly controllable liquid-phase process that does not require expensive equipment [15,16,17,18]. Polyol synthesis typically results in a nanometers-thick residual polyvinylpyrrolidone (PVP) dielectric layer, which covers the NWs [1,2,3,13,17,18]. This layer hinders the current flow between the AgNWs, decreasing the conductivity of the percolating network. A conventional approach to improve the conductivity of such TCCs is the post-synthetic annealing at temperatures ranging from 200 to 400 °C [19,20]. While this step satisfies many applications, such a high temperature processing is not compatible with temperature-sensitive components used in AgNW TCCs, such as polymer substrates and protective coatings. The former is needed for flexible electronics applications, whereas the latter is required to improve the long-term mechanical and chemical stability of AgNWs, which are known to readily oxidize and sulfurize in the surrounding environment [21,22,23,24].

Laser and flash lamp processing, which uniquely enable low-temperature annealing through plasmon welding, are promising alternatives [25,26,27]. However, these techniques require time-consuming raster scanning and show highly material-dependent absorption, as a result of which, samples may suffer from non-uniform heating [28]. Plasma annealing, chemical treatment, and mechanical pressing are non-thermal processes that can be used to improve the electrical performance of AgNW TCCs, but exhibit high processing time, slowing down the roll-to-roll fabrication [29,30,31,32]. In addition, these techniques may damage the polymer substrates (e.g., chemically or mechanically) and are challenging to use if the post-passivation annealing of TCCs is required. The irradiation with continuous ion and electron beams is another low-temperature approach. For example, electron beam annealing was effectively applied to improve the electrical performance of transparent AgNWs networks without degrading the TCC optical properties [33,34,35]. However, continuous beams typically have a relatively low intensity, which, similar to previously discussed techniques, require a high processing time in order to perform the necessary modifications [36,37,38].

In this work, we used an intense pulsed ion beam (IPIB) to improve the conductivity of AgNW TCCs and demonstrated how this approach can be used to improve the performance of transparent heaters, covered with a temperature-sensitive polymer coating. The latter was used to improve the AgNW resistance against oxidative degradation. The IPIB consisted of protons with a peak kinetic energy of 300 keV and a pulse duration of 200 ns, while the beam current density was 30 A/cm^2^. The kinetic energy and sample composition determined the interaction volume (i.e., how deep the heating will take place), while the pulse duration and intensity were responsible for the heating itself. Together, these parameters made IPIBs a promising technique for the low-temperature annealing, since a large number of ions is delivered to the near-surface layer of the target within an ultra-short period of time [39,40,41]. As a result, the surface layer is heated to very high temperatures within an ultrashort period of time, making the overall annealing thermal budget very small. Another unique feature of the IPIB is that the heat epicenter, induced by high current, can be “planted” inside the sample (for example, micrometers deep), from which it will propagate to the surface and the bulk. This enables the annealing of samples, passivated with temperature-sensitive substrates.

## 2. Materials and Methods

Silver nitrate (AgNO_3_, 99.8%), poly-vinyl pyrrolidone (PVP, M_w_ = 360 k), iron chloride (FeCl_2_·6H_2_O, 99.5%), poly-vinyl alcohol (PVA) and ethylene glycol (EG, 99.0%) were received from Sigma-Aldrich (St. Louis, MO, USA). All reagents were used as received without further purification.

### 2.1. Synthesis of Silver Nanowires

AgNWs were synthesized by a modified polyol method. First, 0.4 g of PVP was added to 10 mL of EG in conical flask with a flat bottom. The mixture was stirred (500 rpm) at 120 °C for 30 min to completely dissolve PVP in EG. Then, 200 μL of 20 mM FeCl_2_·6H_2_O dissolved in EG, was added to the mixture. After 5 min, 2 mL of 1 M AgNO_3_ in EG was added dropwise within 10 min. The reaction was allowed to continue for 2 h without stirring. Afterwards, the mixture was consistently washed in ethanol and deionized water by centrifugation at 2000 rpm for 10 min. The final product was dispersed in ethanol for further use.

### 2.2. Fabrication of Transparent Conductive Films

To test a wider range of temperatures, relevant for the electronics powered with 12 V, the AgNWs-based TCCs were fabricated on the surface of the glass. However, it is also possible to fabricate a heater on polymer substrates if lower heater temperatures are required.

Microscopic glass slides (5 × 2.5 cm^2^, ISOLAB GmbH, Eschau, Germany) were used as substrates for AgNWs based TCCs fabrication. The glass slides were cleaned consequently by sonication for 10 min in DI-water and ethanol (Aidabol JSC, Kazakhstan). Next, 1 mL of ethanol dispersion, with as-synthesized AgNWs, was dropped on the glass slides and spin-coated at 2000 rpm for 40 s. The obtained TCCs were dried in the oven at 60 °C for 10 min. The AgNWs spin-coating procedure was repeated five times. PVA solution was prepared by dissolving 1 g of PVA in 20 mL of DI-water while stirring at 90 °C for 3 h. Consequently, the glass slides with AgNWs networks were encapsulated with PVA solution by spin-coating at 2000 rpm for 60 s.

Oxidation stability of TCCs before and after the capping with the PVA protective layer was performed on UV/ozone cleaning system (UVOCS Inc., Lansdale, PA, USA).

The irradiation of AgNWs was carried out on INURA pulsed ion accelerator (Nur-Sultan, Kazakhstan) [42]. As a final step, TCCs were irradiated by a single pulse of IPIB with following parameters: full pulse width is 200 ns, accelerating voltage is 300 kV, beam current density is j = 30 A/cm^2^.

To analyze the performance of TCCs as transparent heaters, two copper strips were attached to the ends of the glass substrate and contacted with the crocodile clips. The remaining area of TCF was 2.5 × 2.5 cm^2^. The potential difference between two copper strips was supplied by BK Precision 1686A DC Power Supply (Yorba Linda, CA, USA).

### 2.3. Characterization

The morphology of AgNWs was analyzed on the Carl Zeiss Crossbeam 540 Scanning Electron microscope, (Oberkochen, Germany). Transmittance spectra of TCFs were measured using the PerkinElmer UV−VIS−NIR spectrophotometer (Waltham, MA, USA). The TCC sheet resistance was measured by a two-probe (two-electrodes) method with BK Precision 1686A DC source and Sanwa PC7000 voltage-meter (Tokyo, Japan). The temperature of transparent heaters was measured with FLUKE Ti300 infrared camera (Everett, WA, USA).

## 3. Theoretical Approaches

**Figure 1:**
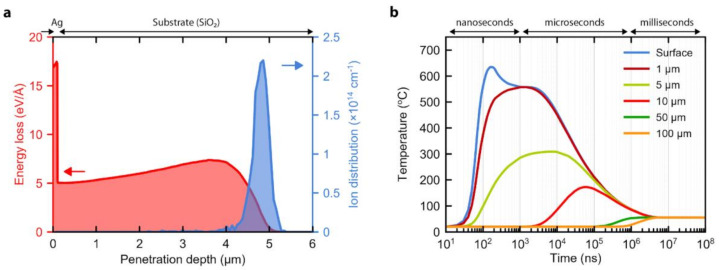
(**a**) Protons energy loss (left, *y*-axis) and distribution (right, *y*-axis) within the sample. (**b**) Intense pulsed ion beam irradiation induced heat transfer in Ag/SiO_2_ structure, showing near-surface annealing capability.

The IPIB interaction with the sample was studied using the Stopping and Range of Ions in Matter (SRIM) software package (SRIM-2013 by James F. Ziegler, Annapolis, MD, USA). For this, a structure consisting of 120 nm thick Ag on SiO_2_ was used, and the proton beam kinetic energy was set to 300 keV, which resembled the actual sample and irradiation parameters. The blue curve in Figure 1a, corresponding to the right *y*-axis, shows protons distribution as a function of penetration depth. The obtained results show that all protons fly through the 100-nm-thick Ag layer and have an average penetration depth of 4.8 μm, thus no AgNWs doping is expected. The red curve in Figure 1a, corresponding to the left *y*-axis, demonstrates the material- and depth-dependent proton energy loss. Although Ag has at least two times higher energy loss, most of the beam energy is being transferred to the substrate. SRIM simulation shows that only the surface layer of the sample interacts with the beam, while the bulk remains non-irradiated.

The energy loss data together with the current density evolution of the proton beam, measured with the Faraday cup, were used to estimate the time- and depth-dependent power density distribution. This function was used as a heat source input in the one-dimensional finite element method analysis, performed using the heat transfer module of the COMSOL Multiphysics software (v 5.4, COMSOL Inc., Stockholm, Sweden). Figure 1b shows the obtained temperature profiles at six different points across the sample: at the surface (i.e., AgNWs temperature) and 1, 5, 10, 50 and 100 μm deep inside the SiO_2_ substrate. As can be seen, the surface of the sample rapidly heats to 635 °C within the first 200 ns, which corresponds to the IPIB pulse duration. The heat originates in the top ~5 μm of the sample within the hundreds of nanoseconds and propagates inside the sample at the microseconds scale with a total annealing time of less than 1 millisecond. The heating and cooling rates were calculated to be 10^10^ and 10^3^–10^8^ °C/s, respectively. The simulations show that the IPIBs induced annealing occurs at the near-surface layer and has an ultra-low thermal budget, both of which are important for the processing of temperature-sensitive materials.

## 4. Results and Discussion

**Figure 2:**
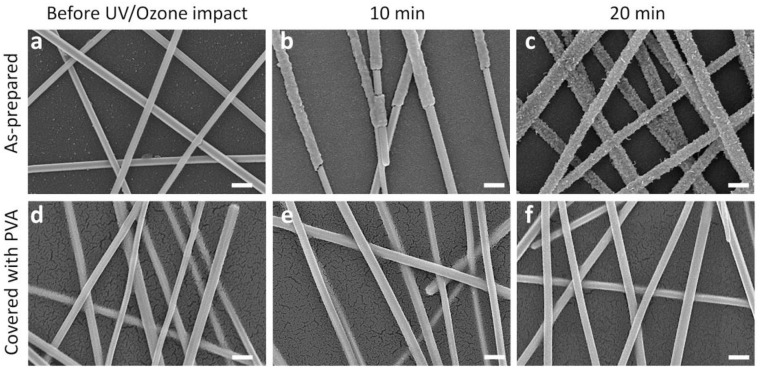
As prepared (non-covered) AgNW transparent conductive coating (**a**) before, and after UV/ozone treatment for (**b**) 10 and (**c**) 20 min. AgNW transparent conductive coating covered with poly-vinyl alcohol protective layer (**d**) before, and after UV/ozone treatment for (**e**) 10 and (**f**) 20 min. All scale bars correspond to 200 nm.

AgNWs are known to have degradation problems, oxidizing and sulfurizing with time if exposed to the ambient [21,22,23,24], which limits their application. To overcome this problem, the TCCs were spin-coated with PVA. The protective coating provides mechanical confinement to the network, enhancing its durability, as well as passivates AgNWs, improving the chemical stability. To test it, the TCCs with and without PVA coating were subjected to UV/ozone treatment for 10 and 20 min. Figure 2 shows the morphology of the resulting structures. As-prepared AgNWs (Figure 2a) have an average diameter of 120 nm, form continuous percolating network and are passivated with a thin layer of PVP (appears white on the SEM image). After a 10 min long UV/ozone treatment (Figure 2b), the oxide shell starts to form around the NWs. After 20 min (Figure 2c) AgNWs are completely covered with a non-uniform rough oxide layer, showing significant degradation. Figure 2d–f shows SEM images before and after UV/ozone treatment of the TCCs covered with PVA. As can be seen, the morphology of the NWs does not change with increasing annealing time, confirming that the PVA coating withstands UV radiation and ozone, as well as prevents the oxygen diffusion and can be used to improve the chemical resistance of TCCs. This demonstration is of special importance for electronics operating in harsh environments.

**Figure 3:**
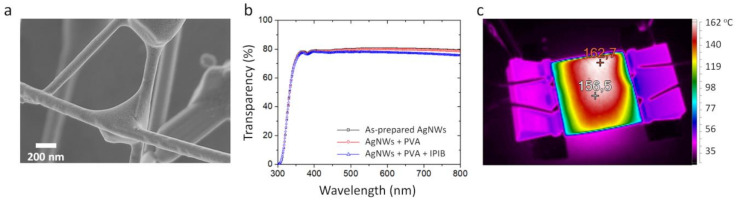
Intense pulsed ion beam (IPIB) modified transparent conductive coating (TCC) covered with poly-vinyl alcohol protective layer (**a**). SEM image of irradiated TCC, (**b**) transmittance spectra of TCC before and after irradiation by IPIB. (**c**) IR-image of TCC based heater.

Next, the electrical performance of AgNW TCCs was evaluated. The as-prepared AgNWs had a resistance of 585 ± 5 Ohm/sq. The increased resistance was caused by the PVP layer on the surface of AgNWs, which hindered the conductivity between individual NWs within the network. After covering AgNWs with PVA, the resistance reduced to 300 ± 15 Ohm/sq, which can be attributed to the formation of a better physical contact between NWs or capillary-force-induced welding caused by the surface tension of the PVA solution. The effective pressure induced by the capillary-forces can reach values as high as 1 GPa, while the mechanical pressure required for AgNWs welding is only 25 MPa [1,30]. To further decrease the resistance of AgNWs covered with PVA, the TCC was annealed by irradiation with a single 200 ns short IPIB pulse with a current density of 30 A/cm^2^, which corresponds to a fluence of 1.5 × 10^13^ protons/cm^2^. Figure 3a shows the SEM image of the resulting structures. After the IPIB impact, PVA slightly redistributed across the network, forming lumps at NW-NW junctions, without exposing the NWs. It follows that the irradiation induced thermal annealing is high enough to soften or melt PVA and short enough to not to evaporate it. The irradiation helped to further reduce the contact resistance down to 100 ± 10 Ohm/sq, which can be explained by additional capillary-force effects and possible annealing-induced welding of NWs. The welding may occur as a consequence of the higher temperature across AgNWs junction, compared to the temperature across the individual NWs. This can be explained by the higher energy loss in Ag, compared to the SiO_2_ substrate (Figure 1a), so a thicker Ag layer results in higher temperature across it.

The transmittance spectra of AgNW TCCs are presented in Figure 3b. All TCCs showed characteristic absorption peaks in the UV-region (300–350 nm), associated with AgNW surface plasmon resonance absorption bands [43,44]. The transmittance of TCC at 550 nm before irradiation was 80%, which after the irradiation changed only slightly across the entire visible range. Thus, the annealing with IPIB resulted in conductivity enhancement without a significant reduction of TCC transparency, which is discussed below.

The fabricated TCCs were tested as transparent heaters, for which a voltage difference of 4, 8, and 12 V was applied across two sides of TCCs, while the temperature evolution was measured using an IR camera. The selected voltage range is suitable for practical applications in low-voltage devices [45,46]. Figure 3c shows a typical IR image of the irradiated AgNW TCC covered with PVA.

**Figure 4:**
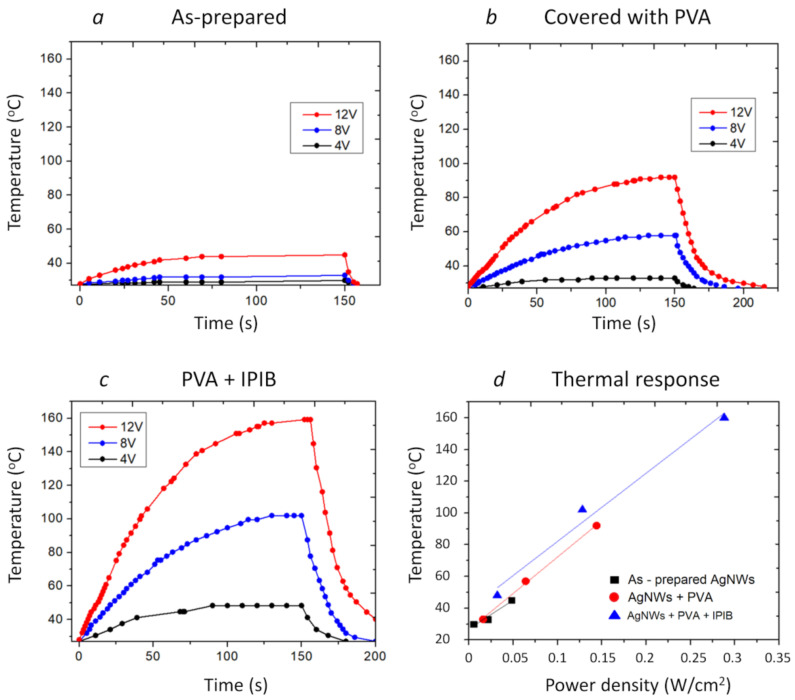
Transparent heater temperature dynamics, monitored by IR-camera, as a function of applied voltage (4, 8 and 12 V) for (**a**) as-prepared AgNWs, (**b**) AgNWs covered with PVA, (**c**) IPIB-irradiated AgNWs covered with PVA. (**d**) Thermal response of TCCs extracted from the temperature dynamics measurements (solid lines represent linear fit of the experimental results).

The IR images, obtained for three types of TCCs (with as-prepared AgNWs, covered with PVA, and after the irradiation), allowed us to observe the Joule heating-induced temperature change and extract the peak temperature. The heater based on the as-prepared AgNWs reached maximum temperatures of 30, 33 and 45 °C at 4, 8 and 12 V, respectively, in 2.5 min (Figure 4a). The heater coated with PVA showed better performance due to improved TCC conductivity (Figure 4b). Maximum temperatures of 33, 57 and 92 °C were achieved applying 4, 8 and 12 V, respectively. After IPIB modification, the performance of the heater further improved (Figure 4c) and the temperatures of 48, 99 and 160 °C were achieved for three voltages used. The thermal efficiency of the fabricated heaters was estimated by extracting the slope of the linear fit of the “temperature—power density” curves, depicted in Figure 4d. After coating with PVA and IPIB irradiation, the slopes were 458 and 430 °C·cm^2^/W, respectively, which is comparable or better than the results reported for other heaters [45,46,47]. Optimization of the synthesis parameters can further improve the optical and electrical performance of heaters by, for example, improving the AgNW aspect-ratio.

## 5. Conclusions

To summarize, we demonstrated that irradiation with IPIB can be used to anneal AgNWs based TCCs, covered with temperature-sensitive PVA protective layers. The super-fast IPIB annealing with a current density of 30 A/cm^2^ reduced the resistance of AgNWs percolating network without degrading the TCC transparency. As a result, the transparent heater’s operating temperature increased from 92 to 160 °C at an applied voltage of 12 V, relevant for anti-fogging or de-icing applications. Compared to other known techniques used for annealing, the irradiation with IPIBs does not suffer from prolonged scanning, offering super-fast annealing on a very large scale.
